# The NIMA-family kinase, Nek1 affects the stability of centrosomes and ciliogenesis

**DOI:** 10.1186/1471-2121-9-29

**Published:** 2008-06-04

**Authors:** Mark C White, Lynne M Quarmby

**Affiliations:** 1 Department of Molecular Biology & Biochemistry, Simon Fraser University, Burnaby, Canada

## Abstract

**Background:**

Mutations in Nek1 (NIMA-Related Kinase 1) are causal in the murine models of polycystic kidney disease *kat *and *kat*^2*J*^. The Neks are known as cell cycle kinases, but recent work in protists has revealed that in addition to roles in the regulation of cell cycle progression, some Neks also regulate cilia. In most cells, cilia are disassembled prior to mitosis and are regenerated after cytokinesis. We propose that Neks participate in the coordination of ciliogenesis with cell cycle progression. Mammalian Nek1 is a candidate for this activity because renal cysts form in response to dysfunctional ciliary signalling.

**Results:**

Here we report that over-expression of full-length mNek1 inhibited ciliogenesis without disrupting centrosomes in the murine renal epithelial cell line IMCD3. In contrast, over-expression of the kinase domain with its associated basic region, but without the acidic domain, caused loss of centrosomes. As expected, these cells also failed to grow cilia. Both defective ciliogenesis in response to too much mNek1 and disassembly of centrosomes in response to expression of the kinase lacking the presumptive regulatory domain was abrogated by kinase-inactivating mutations or by removal of the coiled-coil domain. We observed that kinase-inactive, C-terminal truncations of mNek1 retaining the coiled-coil domain localized to the cilium, and we define a ciliary targeting region within the coiled-coil domain.

**Conclusion:**

Based on our data, we propose that Nek1 plays a role in centrosome integrity, affecting both ciliogenesis and centrosome stability.

## Background

NimA-R elated Kinase 1 (Nek1) is a member of the kinase family sharing sequence identity to catalytic domain of the essential *Aspergillus nidulans *gene *nimA *(Never In Mitosis A; [1]). Mutations in Nek1 are causal in the murine models of polycystic kidney disease *kat *and *kat*^2*J*^, resulting in a recessive pleiotropic phenotype that includes progressive cystic kidneys, male sterility, dwarfing, abnormal olfactory lobes, facial dysmorphism, choroid plexus cysts, hydrocephalus, uremia and anemia [2-4]. Although it has been suggested that Nek1 may play roles in cell-cycle control, meiosis, gametogenesis, and in the nervous system [1,5] the cellular function of Nek1 has remained elusive.

The Neks are considered to be cell cycle kinases [6], but some members of the family play additional roles in ciliary function. Our studies in *Chlamydomonas *revealed both cell cycle and ciliary functions for two Neks: Fa2p plays a role at the G_2_/M transition of the cell cycle and is essential for the deflagellation response [7,8], whereas Cnk2p is a ciliary protein that participates in the assessment of cell size prior to commitment to enter mitosis and plays a role in regulation of flagellar length [9]. A survey of the Neks encoded by the genomes of various organisms supported our idea that the Nek family may have co-evolved with the alternating use of centrioles as basal bodies and as the foci of spindle poles [10]. These results lead us to speculate that dual roles in ciliary and cell cycle regulation might be a hallmark of the Nek family.

We recently completed a comprehensive phylogenetic analysis of the Nek family across the eukaryotes and discovered that they are an ancient family [11]. Our analyses suggest that six or seven different Neks were already present in the ciliated unicellular organism that gave rise to all of the eukaryotic lineages. The further observation that Neks representative of each extant clade localize to centrosomes and/or cilia supports our idea that the original expansion of the Neks was associated with dual roles in the regulation of cilia and the cell cycle, possibly co-ordinating ciliogenesis with cell cycle progression [11]. Dual roles in cilia and cell cycle have not yet been demonstrated for any of the mammalian Neks.

There is some indication that the mammalian kinase, Nek1 may subserve both roles. Aberrant ciliary signaling and cell cycle regulation are both implicated in the etiology of renal cysts [12,13] and mutations in Nek1 are causal for kidney cyst formation. Additionally, we have previously localized Nek1 to centrosomes [14]. We thus set out to explore the cellular activities of Nek1 in order to further test our hypothesis about the dual roles of this kinase family.

Here, we report that transient over-expression of mNek1 in the ciliated renal epithelial cell line IMCD3 results in failure to assemble cilia. This effect depends upon the kinase activity of Nek1. In contrast, expression of dominant negative forms of Nek1 caused disruption of centrosomes, as indicated by loss of γ-tubulin and centrin foci. These disruptive effects require the coiled-coil domain of Nek1, in addition to kinase activity. We show that the coiled-coil domain of Nek1 is sufficient for nuclear, centrosomal and ciliary localization of the protein. We propose that Nek1 activity in differentiated kidney epithelial cells contributes to the dynamic regulation of the centrosome and the primary cilium, a sensory apparatus critical for the maintenance of tubules and in the prevention of renal cytogenesis.

## Results

### mNek1 is comprised of a basic region and an acidic region

**Figure 1:**
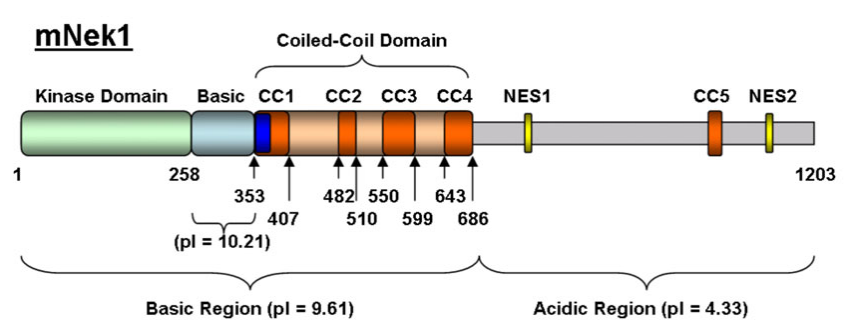
**Schematic representation of mNek1 depicting major features of the primary structure of the protein**. Numbers indicate amino acid position in the primary sequence of mNek1 (mRNA, AY850065; protein, AAB23529). Major features include: N-terminal kinase domain (light green, residues 1–258), a basic domain (pI = 10.21, light blue, residues 258–353), a coiled-coil domain (light orange, residues 353–686) containing four coiled-coils (red, CC1–CC4, residues indicated), a fifth coiled-coil in the C-terminus (red, CC5), a predicted NLS (blue, residues 355–378) and two predicted NESs (yellow, 764–774 and 1131–1138). Although the full-length sequence of mNek1 has a predicted isoelectric point of 5.45, the sequence can be divided into a basic N-terminus (Basic Region, pI = 9.61, residues 1–686) and an acidic C-terminus (Acidic Region, pI = 4.33, residues 686–1203).

Figure 1 illustrates the predicted domain structure of murine Nek1 (mNek1), highlighting important features mentioned in this text. Using the isoelectric point function of Protein Calculator (created by Dr. Christopher Putnam, The Scripps Research Institute, La Jolla, CA) we observe that mNek1 can be conceptually divided into a basic N-terminus (residues 1–686) and an acidic C-terminus (residues 687–1203). The N-terminal kinase domain (residues 1–258) is 96% identical (98% similar) to human Nek1. The coiled coils are predicted by COILS v2.2 [15] using a > 0.6 score in one of the three window lengths (most of the coiled-coil is > 0.995 in at least one of the windows, the > 0.6 score is used to define the boundaries). A single NLS at residues 355–378 of mNek1 is predicted by the nuclear localization signal prediction program PredictNLS [16]. Two leucine-rich nuclear export sequences (NESs), NES1 at aa 764–774 and NES2 at aa 1131–1138, are predicted by the nuclear export sequence (NES) prediction program NetNES v1.1 [17].

### Transfected constructs are first expressed prior to post-mitotic ciliation

**Figure 2:**
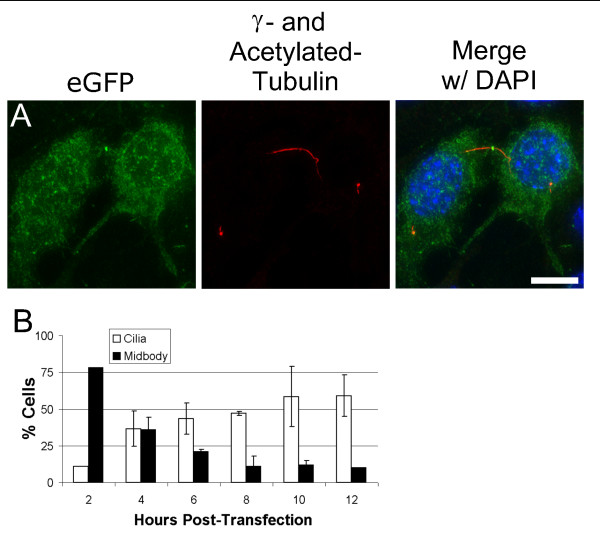
**Transiently transfected constructs are first expressed as cells exit mitosis**. (A) Representative fluorescence microscopy image of IMCD3 cells expressing eGFP (green) two hours post-transfection connected by a cytokinetic bridge stained with acetylated-tubulin (red). (B) Graph of the presence of cytokinetic structures and primary cilia over a post-transfection time course. Error bars represent the standard deviation of two experiments, n = 100 for each. The high percentage of transfected cells possessing cytokinetic structures two hours post-transfection indicates that these cells recently completed mitosis. Bar, 10 μm.

Entry of a transfected plasmid into the nucleus to gain access to the transcription machinery is one of the critical steps limiting exogenous gene expression [18]. Liposome-mediated transient transfection is greatly enhanced by events associated with mitosis [19,20], probably due to the nuclear encapsulation of plasmid arising from the breakdown and reformation of the nuclear envelope [21]. Two hours post-transfection with pEGFP, we observe that ~80% of IMCD3 cells expressing eGFP are adjacent to another eGFP-expressing cell, often still connected by a cytokinetic bridge (detected with an acetylated-tubulin antibody; Figure 2A). We infer that under our experimental conditions, cells first express exogenous protein following mitosis. Thus, as previously reported by others, recently-transfected cells expressing the exogenous protein represent a population having recently completed mitosis.

Cells reabsorb their cilia prior to mitosis and regrow them after mitosis (reviewed in [22]). If the only cells expressing eGFP in the first few hours after transfection are those that have recently completed mitosis, then we predicted that the extent of ciliation of this population of cells will increase with time, corresponding to post-division ciliogenesis. As expected, ciliation of eGFP-expressing cells increases between 2 and 12 hours post-transfection (see Figure 2B).

### Transient over-expression of eGFP-mNek1 disrupts ciliogenesis

**Figure 3:**
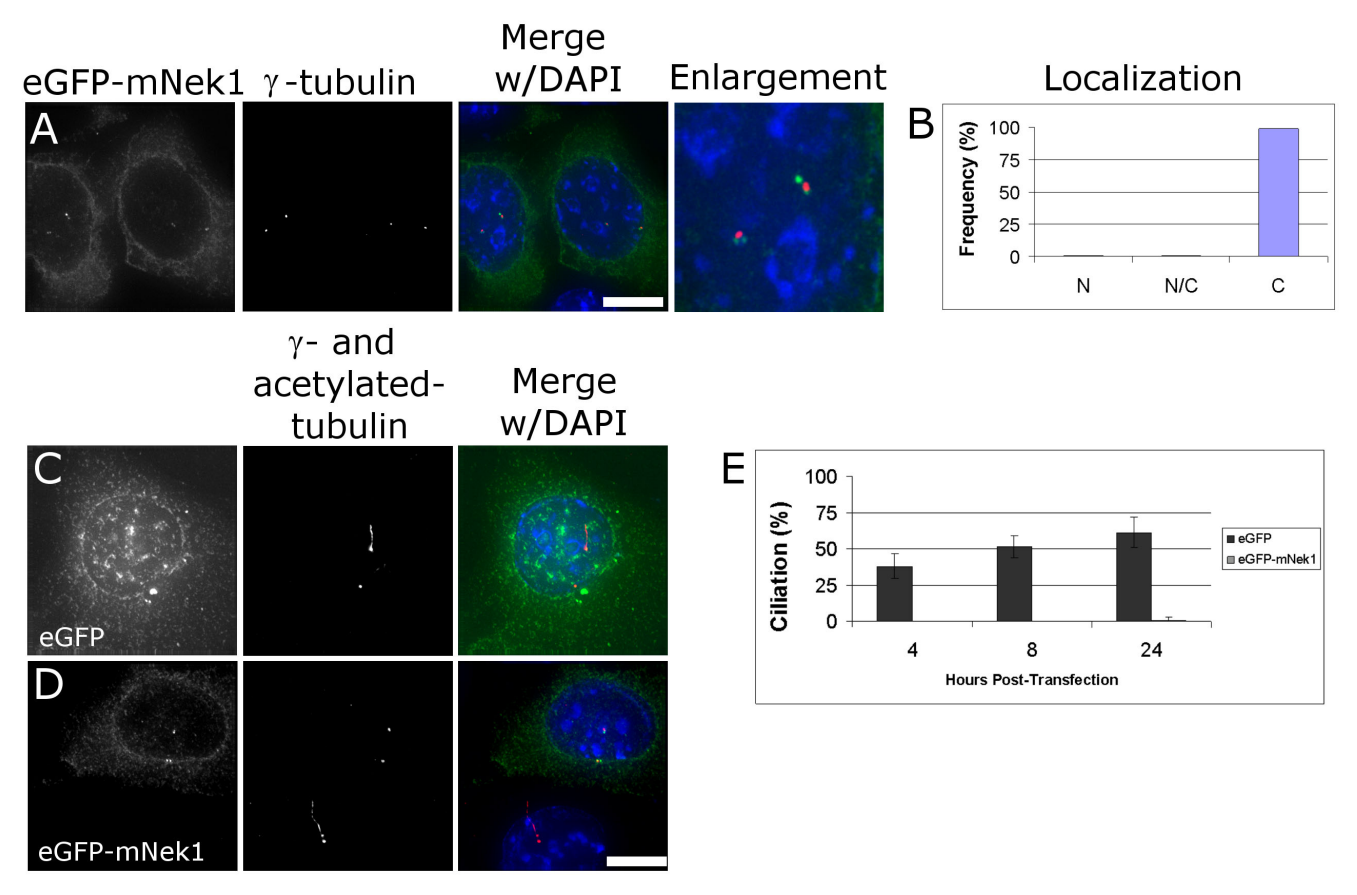
**Transient overexpression of mNek1 disrupts ciliogenesis**. (A) eGFP-mNek1 (green) localizes to several spots adjacent to the γ-tubulin foci (red) and is excluded from the nucleus (DAPI, blue). (B) Nuclear localization was scored as either predominantly nuclear ("N"), approximately equivalently distributed between nucleus and cytoplasm and nucleus ("N/C"), or predominantly cytoplasmic ("C"), n = 300. (C) eGFP (green) is not found associated with centrosomes and is observed in both cytoplasm and nucleus. These cells were fixed and stained 12 hours after transfection. Note the presence of a full-length primary cilium. (D) Representative fluorescent image of eGFP-mNek1 transfected cell lacking a primary cilium 12 hours after transfection. Note the ciliated wild-type cell in the same image. (E) Quantification of cells expressing a primary cilium at 4, 8 and 24 h post transfection. Error bars represent the standard deviation between two experiments, n = 200 for each experiment. Bar, 10 μm.

Subcellular localization of transiently transfected mNek1 N-terminally tagged with eGFP matches previous reports of localization of the endogenous mNek1 [14]. That is, eGFP-mNek1 (green) localizes to foci adjacent to the γ-tubulin-rich centrosomes (red) and is excluded from the nucleus (Figure 3A, B). We observe that IMCD3 cells transfected with eGFP-mNek1 for 4, 8 or 24 hours lack the primary cilium (Figure 3D, E) but retain γ-tubulin-rich centrosomes (Figure 3A, D). In contrast, cells transfected with eGFP alone are increasingly ciliated over 4, 8 and 24 hours (Figure 3C, E). These data indicate that over-expression of mNek1 inhibits ciliogenesis.

In order to determine whether inhibition of ciliogenesis requires kinase activity, we generated the mutation K_33_R in the invariant lysine residue within the ATP-binding domain [23]. The cognate mutation inactivates kinase function in Nek2 and Nek11 [24,25]. We refer to constructs containing this mutation as presumptive kinase-dead. In contrast to cells expressing mNek1 with a lysine at position 33, cells expressing mNek1 containing the kinase-inactivating K_33_R mutation show no abnormal phenotype (data not shown).

### Deletion of the presumptive regulatory acidic domain creates a dominant negative form of Nek1 which disrupts centrosomes

Several kinases, such as FAK and Plk, are regulated in part through inhibition of kinase activity by the C-terminal, non-kinase domain of the protein [26,27]. The C-terminus of Nek1 can bind the N-terminal kinase domain [28], and we sought to determine whether the acidic C-terminal domain of mNek1 influenced the ability of overexpressed eGFP-mNek1 to disrupt the primary cilium.

IMCD3 cells were transiently transfected with various truncations of eGFP-tagged mNek1 and those expressing eGFP were scored for the presence of centrosomes (γ-tubulin foci) and cilia (Table 1). Neither eGFP alone, nor the eGFP-tagged kinase domain of mNek1 (residues 1–258) affected centrosomes or cilia (Figures 3C and Table 1), but expression of the eGFP-tagged basic region (which includes the kinase domain; residues 1–686) resulted in the loss of centrosomes in a substantial proportion of cells (Table 1). Given that the integrity of centrosomes is a pre-requisite for ciliogenesis, it is not surprising that these cells also lack cilia. Cells expressing an eGFP-N-terminal mNek1 fusion protein (residues 1–352 only) retain their γ-tubulin foci and their cilia, indicating that disruption of centrosomes requires the coiled-coil domain present in residues 353–686. With the exception of the kinase domain alone these truncations localize to the nucleus, an observation in accord with a previous report [29].

**Table 1:** Expression of mNek1 truncations disrupt centrosomal γ-tubulin.

**mNek1 aa residues expressed with GFP tag**	**Subcellular localization of GFP-truncated Nek1**	**Percent of transfected cells without γ-tubulin foci**
1–258	Cytoplasmic	0
1–352	Nuclear	0
1–407	Nuclear	8 ± 1
1–510	Nuclear	68 ± 3
1–599	Nuclear	64 ± 3
1–686	Nuclear	64 ± 4
1–686 (K33R)	Ciliary	0

Quantification of the loss of γ-tubulin foci is based on examination of 100 transfected (green) cells from each of three independent experiments. These were scored as either retaining or lacking detectable γ-tubulin foci. Shown is the average number of transfected cells that lack γ-tubulin foci, plus or minus the standard deviation. Constructs containing at least the first two coiled-coils significantly reduce the number of transfected cells retaining γ-tubulin foci (constructs 1–510 and 1–599).

In order to determine which of the predicted coiled-coils is necessary for the depletion of γ-tubulin foci, we expressed a panel of eGFP-tagged, truncated mNek1 constructs that sequentially remove the four coiled-coils of the coiled-coil domain. We observe that the constructs containing both coiled-coils 1 and 2 (residues 1–510), the first three coiled-coils (residues 1–599), and all four (residues 1–686) cause depletion of γ-tubulin foci, whereas the constructs containing either none (residues 1–352), or only the first coiled-coil (residues 1–407) fail to deplete γ-tubulin foci. These data suggest that the second coiled-coil of mNek1 (possibly in combination with the first coiled-coil) is required for the depletion of γ-tubulin foci, whereas the third and forth coiled-coils are not involved in this activity.

**Figure 4:**
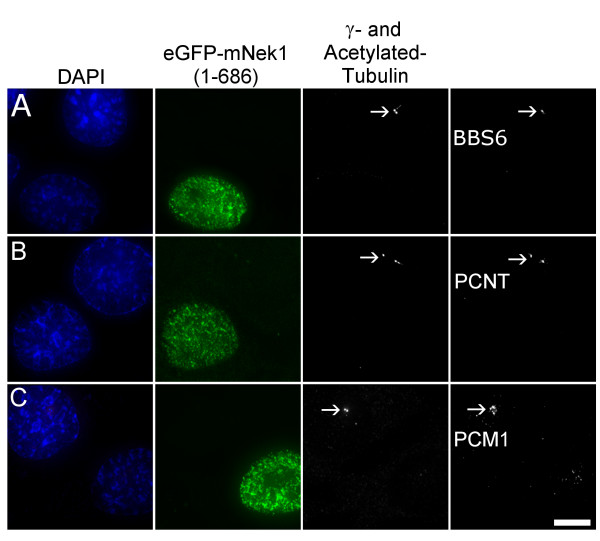
**Pericentriolar disruption resulting from overexpression of mNek1 (1–686) is not limited to γ-tubulin**. (A-C) Representative fluorescence microscopy images of IMCD3 cells expressing eGFP-mNek1 (1–686), co-stained with acetylated- and γ-tubulin and BBS6 (A), Pericentrin (PCNT; B) or PCM1 (C). Transfected cells lack foci of these centrosomal markers. The location of the intact centrosome in the non-transfected cell is marked by an arrow. Bar, 10 μm.

In order to determine whether disruption of centrosomes requires kinase activity, we generated the presumptive kinase-dead K_33_R mutation in the various truncation constructs. In contrast to cells expressing truncated forms of mNek1 with a lysine at position 33, cells expressing truncations of mNek1 containing the kinase-inactivating K_33_R mutation retain their γ-tubulin foci and their cilia (Table 1). We conclude that kinase activity is required for disruption of centrosomes. In addition, these cells lacked foci of the centrosomal components pericentrin, PCM1, BBS6 and the targets of autoimmune serum M4491 (Figure 4, and data not shown). This indicates that the effect represents the loss of pericentriolar material, and is not specifically limited to γ-tubulin.

### γ-tubulin loss occurs rapidly after expression of mNek1 and precedes centrin loss

**Figure 5:**
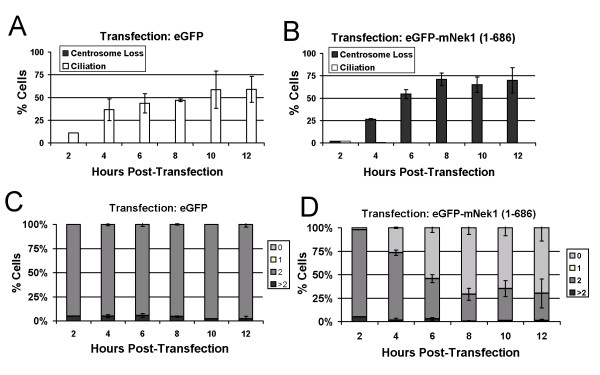
**mNek1 disruption of centrosomes is not due to sequential loss**. Cells were scored for the presence or absence of cilia and centrosomes following transfection with eGFP-alone (A) and eGFP-mNek1 (1–686) (B). Error bars represent standard deviation of 3 experiments, n = 100 for each experiment. (C and D) Time course of γ-tubulin foci number (0, 1, 2 or > 2) in cells expressing eGFP alone (C) or eGFP-mNek1 (1–686) (D). Cells transfected with eGFP-mNek1 (1–686) are not observed to retain a single γ-tubulin focus, suggesting that loss is coincident.

To evaluate the kinetics of loss of the pericentriolar material of eGFP-mNek1 (1–686), we examined cells during the first 12 hours post-transfection. In contrast to eGFP-transfected cells (Figure 5A), which displayed increasing numbers of ciliated cells and retention of γ-tubulin foci, eGFP-mNek1 (1–686) transfected cells (Figure 5B) lost their γ-tubulin foci (and failed to assemble cilia) within the first four hours post-transfection. Additionally, we never observed a cell transfected with eGFP-mNek1 (1–686) retain exactly one γ-tubulin focus during the first 12 hours post-transfection (Figure 5D). Because the loss of detectable γ-tubulin foci occurs within a few hours, and because we did not observe cells with only one γ-tubulin focus, we infer that the loss of pericentriolar material is not a consequence of defects in centriolar replication or segregation of centrosomes during division.

**Figure 6:**
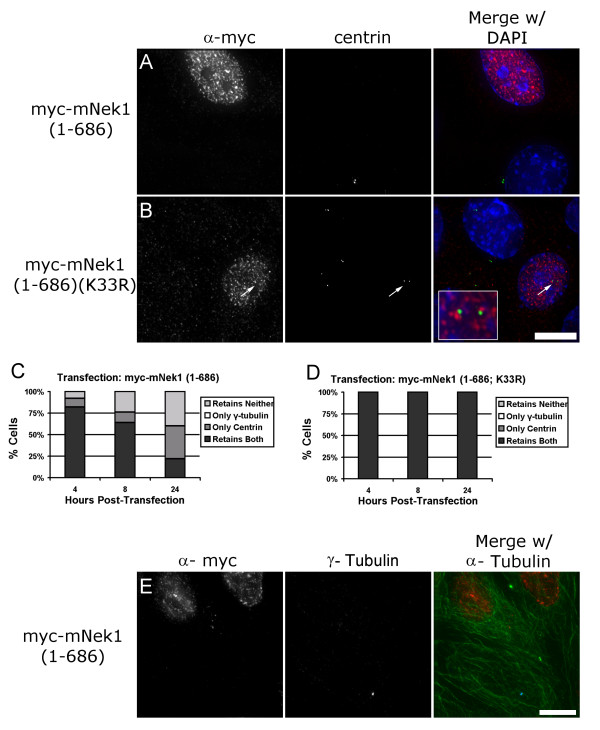
**Overexpression of mNek1 (1–686) causes the dismantling of the centrosome**. Expression of myc-mNek1 (1–686) (red, A) results in loss of centrioles (centrin, green), whereas myc-mNek1 (1–686) (K_33_R) transfected cells retain centrin foci (B). (C and D) Time course of γ-tubulin foci and/or centrin foci presence in cells transfected with (C) myc-mNek1 (1–686), or (D) myc-mNek1 (1–686; K_33_R). Cells transfected with myc-mNek1 (1–686) are not observed to retain γ-tubulin foci while lacking centrin foci, indicating that loss of γ-tubulin foci precedes loss of centrin. (E) Representative image of α-tubulin (green), myc-mNek1 (1–686) (red), and γ-tubulin (blue) demonstrating that gross alterations to the microtubule cytoskeleton do not precede γ-tubulin loss in transfected cells. Bar, 10 μm.

In order to determine whether mNek1 affected the stability of the centrioles in addition to the pericentriolar proteins, we stained for centrin, a centriolar component that localizes to the distal centriolar lumen [30]. During the first 4- to 24-hours post transfection an increasing fraction of cells transfected with myc-mNek1 (1–686) lack centrin-containing foci (Figure 6A, C), whereas cells transfected with the presumptive kinase-dead construct myc-mNek1 (1–686, K_33_R) retain centrin foci (Figure 6B, D). Co-staining with γ-tubulin revealed instances of cells with centrin foci devoid of γ-tubulin, but we never observed a cell that retained γ-tubulin foci that lacked centrin (Figure 6C). These observations indicate that γ-tubulin loss precedes centrin loss. To determine whether gross detrimental effects to the cytoskeleton precede loss of γ-tubulin, we visualized cellular microtubules with an α-tubulin antibody. We did not observe changes in the microtubule network, even in cells that lack γ-tubulin foci (Figure 6E), leading us to conclude that effects on the cytoskeleton did not cause the loss of γ-tubulin foci.

**Figure 7:**
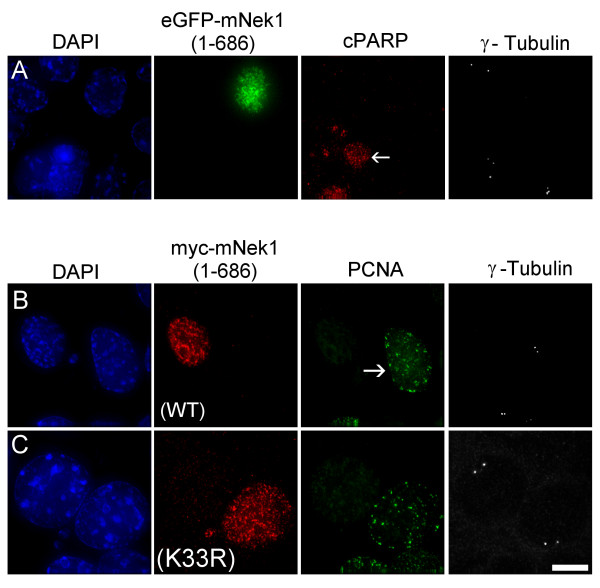
**mNek1-mediated centrosomal loss disrupts the cell cycle and is not a consequence of apoptosis**. (A) Representative fluorescence microscopy image of IMCD3 cells transfected with eGFP-mNek1 (1–686; green), co-visualized with the apoptosis marker cleaved-PARP (red), γ-tubulin (greyscale) and DAPI (blue). Adjacent to an untransfected, representative apoptotic cell (arrow), the transfected cell lacks γ-tubulin foci but remains negative for cleaved-PARP. (B and C) Representative fluorescence microscopy images of IMCD3 cells transfected with either (B) myc-mNek1 (1–686) or (C) myc-mNek1 (1–686; K_33_R). α-myc (red) is co-visualized with the S phase marker PCNA (green), γ-tubulin (greyscale) and DAPI (blue). An untransfected, representative S phase cell is indicated with an arrow. 24 hours post-transfection, cells transfected with myc-mNek1 (1–686) that lack γ-tubulin foci are not observed in S phase, whereas 10% of cells transfected with myc-mNek1 (1–686; K_33_R) are positive for PCNA.

We next asked whether the disruptive forms of mNek1 were triggering loss of centrosomes as a consequence of causing activation of an apoptotic program. Using an antibody against cleaved-PARP as a marker for early apoptosis, we determined that cells lost their γ-tubulin foci and yet remained negative for cleaved-PARP (Figure 7A). Furthermore, cleaved-PARP positive cells are no more common in cultures transfected with disruptive constructs than in cell transfected with non-disruptive mNek1 constructs (data not shown). We conclude that loss of γ-tubulin foci is not a consequence of apoptosis.

To evaluate whether transfected cells with compromised centrosomes continue to progress through the cell cycle, we co-stained with an antibody against PCNA (proliferating cell nuclear antigen), an accessory protein for polymerase δ often used as a marker for cells in S phase [31]. At 24 hours post-transfection with myc-mNek1 (1–686), we observe that transfected cells lacking γ-tubulin foci are negative for PCNA staining, whereas 10% of myc-mNek1 (1–686, K_33_R) expressing cells are positive for PCNA (Figure 7B, C). We conclude that mNek1-induced centrosomal loss results in failure to progress through the cell cycle. This is consistent with a recent report that the integrity of the centrosomes is a requirement for progression through the cell cycle [32].

### Presumptive Kinase-dead mNek1 localizes to the primary cilium

**Figure 8:**
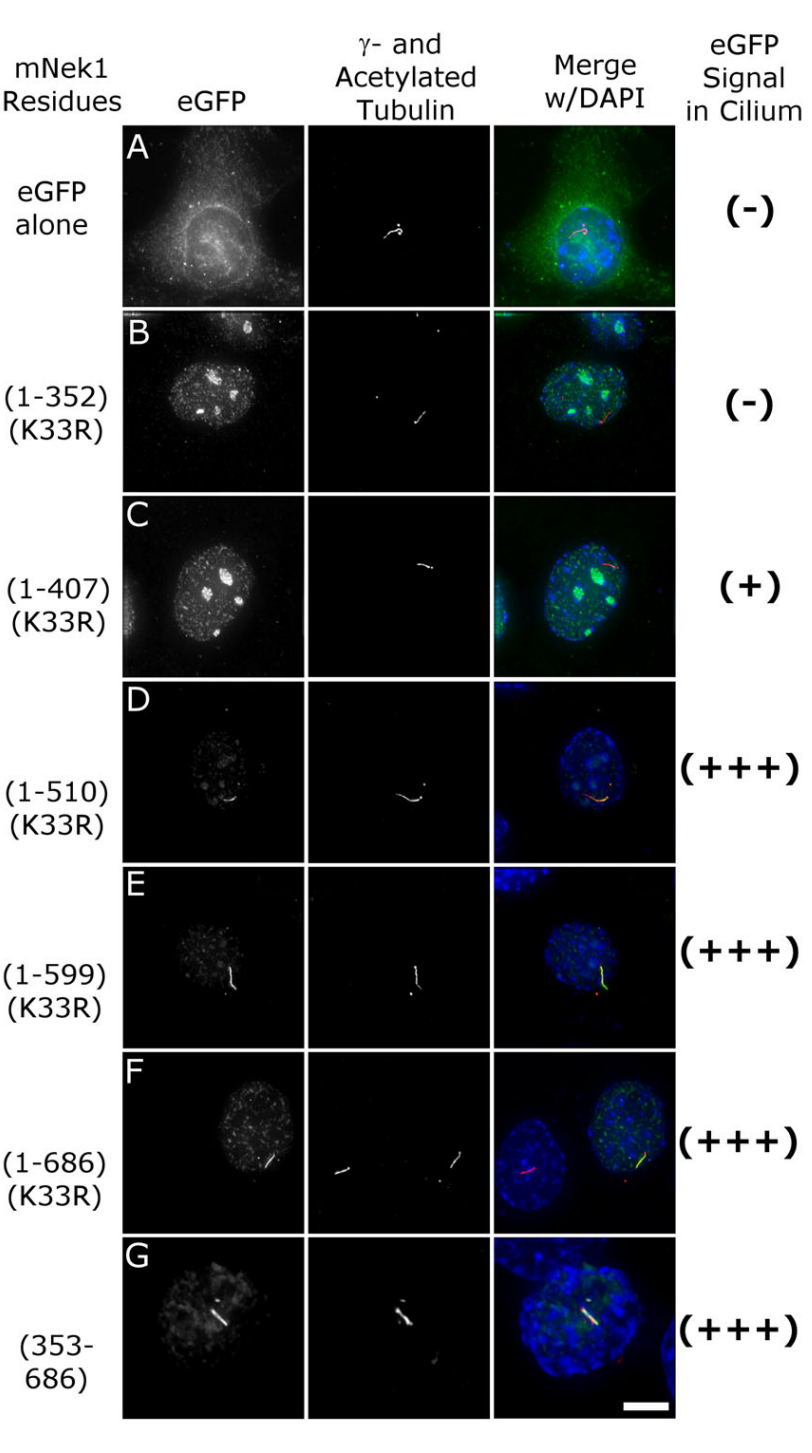
**The Coiled-coil domain of mNek1 contains a ciliary localization signal**. (A-G) Representative images of eGFP-tagged mNek1 constructs containing the mutation K_33_R (green), γ- and acetylated-tubulin (red), and a merge with DAPI (blue). Localization to the primary cilium was scored by the co-localization of the acetylated-tubulin-stained cilium and the eGFP signal as either none (-), minimal (+), or strong (+++). Constructs that contain coiled-coil 2 localize to the primary cilium (D-G), and the coiled-coil domain is sufficient for localization to the primary cilium (G). Cells in these images were fixed and stained 12 hours after transfection. Bar, 10 μm.

Intriguingly, we observed ciliary and centrosomal localization of presumptive kinase-dead (K_33_R) versions of the same Nek1 full-length and truncation constructs that caused inhibition of ciliogenesis and disruption of centrosomes, respectively (Figure 8 and data not shown). These data suggest that the disassembly of centrosomes induced by kinase-active forms of mNek1 requires correct targeting to these organelles.

Expression of a panel of constructs that encode fusion proteins removing the four coiled-coils revealed that constructs containing coiled-coils 1 and 2 localize to the primary cilium (compare Figure 8C and 8D) whereas coiled-coils 3 and 4 are dispensable for localization to the primary cilium (compare Figure 8D–F). Furthermore, the coiled-coil domain alone is sufficient for localization to the primary cilium (Figure 8G).

The observation of Nek1 localization to the primary cilium is novel. We have not observed endogenous mNek1 in the primary cilium (data not shown; [14]), nor do we observe any of our other tagged-Nek1 constructs in the cilium. Based on these observations, we propose that Nek1 may normally cycle through the ciliary compartment but only kinase-dead Nek1 is retained in the cilia long enough (or in sufficient quantities) to be visualized.

## Discussion

Nek1 is a centrosome-associated protein and mice without functional Nek1 develop polycystic kidneys [2-4]. A link between centrosomal or ciliary dysfunction and the formation of kidney cysts is well-established and generally thought to be causal, although the molecular details have not yet been worked out [12,13]. Kidney cysts are associated with aberrant cell proliferation, differentiation, apoptosis and loss of proper orientation of mitotic spindles along the axis of the renal tubules [33-37]. This is consistent with a growing body of evidence that many signals for proliferation and differentiation are received and processed by the primary cilium [38,39]. Consequently, Nek1 provides a promising opportunity to test our prediction that some mammalian Nek proteins play dual roles in the regulation of cilia and cell cycle progression [10,22].

We have shown here that transient over-expression of mNek1 inhibits ciliogenesis and overexpression of a Nek1 construct lacking its inhibitory domain causes disassembly of centrosomes. These data suggest that the normal function of the endogenous protein might involve regulation of centrosomal integrity and ciliogenesis. Unfortunately, we were unable to complement this work with studies of mNek1 knock-down because none of our mNek1 RNAi experiments yielded greater than 50% knock-down of mNek1 protein expression, and we did not detect any mutant phenotypes in these cells (data not shown). It is unlikely that this failure to generate knock-down cells is a consequence of some essential function of mNek1 because the *kat *and *kat2J *mice are viable as homozygotes. Nevertheless, the over-expression studies allow us to deduce some aspects of the cellular activities of Nek1.

We found that overexpression of full-length Nek1 and certain truncated forms of the protein inhibit ciliogenesis (see Figures 3 and 5). We think that the over-expression phenotype is a consequence of excessive mNek1 kinase activity. This conclusion is supported by the observation that over-expression of presumptive kinase-dead forms of mNek1 do not inhibit ciliogenesis. Over-expression of full-length kinase is expected to add substantial Nek1 kinase activity to the cell, even if the bulk of the protein is inhibited. An excess of Nek1 activity could inhibit ciliogenesis by blocking post-mitotic upregulation of ciliary proteins, by interfering with assembly or docking of pre-ciliary complexes at the centrosome, or by stimulating excessive rates of ciliary disassembly.

There are precedents for Nek activity to enhance rates of ciliary disassembly. Ciliary assembly, disassembly and length control are all mediated by a balance of the rates of anterograde and retrograde IFT (intraflagellar transport; [40]). The *Chlamydomonas *Nek Cnk2 is an axonemal protein that affects ciliary length by modulating the rate of disassembly and the delay before onset of assembly [9]. Stable overexpression results in cilia that are shorter than normal, whereas RNAi-based knockdown results in long cilia. The shorter flagella were shown to be a consequence of accelerated rates of disassembly in the *Chlamydomonas *cells that were overexpressing Cnk2. Importantly, the longer cilia in cells with reduced Cnk2 were not a consequence of decreased rates of disassembly. Rather, when levels of Cnk2 are reduced there is a decrease in the lag time preceding ciliary assembly. Taken together, these data suggest that activated Cnk2 promotes ciliary disassembly both during reabsorption of cilia and in the initial stages of ciliogenesis, perhaps providing a ciliary assembly checkpoint which coordinates ciliogenesis with exit from mitosis. In the multi-ciliated protist *Tetrahymena*, overexpression of the Cnk2 orthologs also result in short cilia, suggesting conservation of function [41]. Nek1 may directly regulate the IFT machinery because it directly interacts with Kif3a, a subunit in the heterotrimeric molecular motor kinesin-2 responsible for anterograde IFT [28].

Overexpression of the mitotic kinase Aurora A leads to the loss of cilia in mammalian cells [42]. Although the disappearance of cilia was attributed to disassembly, inhibition of post-mitotic ciliogenesis was not ruled out. Nevertheless, loss of cilia in the context of excess Aurora A was shown to be mediated by activation of the tubulin deacetylase HDAC6. Pugacheva and colleagues showed that cilia are retained in cells with excess Aurora A, if these cells are treated with HDAC inhibitors. In contrast, we could not rescue Nek1-dependent cilia loss by incubation with the same HDAC inhibitors, although acetylation of microtubules was greatly increased in these cells (data not shown). We conclude that Nek1 is affecting ciliogenesis by a different pathway than Aurora A.

We next considered the possibility that Nek1 was affecting ciliogenesis as a consequence of disruptive effects on the centrosomes/basal body apparatus, the docking site for protein complexes en route to the cilium. We did not detect disruption of localization of centrosomal components in cells overexpressing Nek1 (Figure 3 and data not shown), nevertheless these cells failed to assemble cilia. It is possible that there may have been subtle centrosomal defects not detected because when truncation mutants lacking the presumptive inhibitory C-terminal domain were expressed, we detected rapid loss of γ-tubulin at the centrosomes, followed by loss of the centrin signal (Table 1 and Figures 5 and 6). Similar to the inhibition of ciliogenesis by the full-length protein, these effects depended upon the kinase activity of Nek1 because constructs carrying the kinase-disrupting mutation K_33_R did not disrupt centrosomal integrity and ciliogenesis occurred normally (Table 1 and Figure 6). We interpret these data to reflect a more severe augmentation of Nek1 kinase activity by the truncation mutants, which may be constitutively active. If the centrosome disassembly induced by these mutant forms of the protein reflects a pathological manifestation of a more subtle centrosome-remodelling activity of the endogenous protein, then these data would support the idea that the inhibition of ciliogenesis by over-expression of full-length mNek1 might be secondary to effects on the integrity of the centrosome.

We observed that presumptive kinase-dead Nek1 truncation mutants localized to the cilium and the nucleus (Figure 8). Kinase-active proteins were also found in the nucleus, but these cells did not have cilia (Table 1). We used presumptive kinase-dead constructs to identify a ciliary targeting domain in the first two coiled-coils of mNek1 (Figure 8). This same domain is also required for the centrosome/primary cilium disruption caused by expression of constructs that encode the wild-type kinase domain, suggesting that Nek1 might act directly on the centrosome and possibly also within the cilium. One caveat is that although the coiled-coil domain of mNek1 is sufficient for localization to the primary cilium, fusion proteins composed of the entire C-terminus of mNek1 (residues 258–1203) fail to localize to the primary cilia (not shown). This could indicate that the C-terminus prevents ciliary accumulation. Although we demonstrate the necessity of a functional ciliary targeting sequence for inhibition of ciliogenesis (Table 1 and Figure 8), we can not rule out the possibility that the effect was via nuclear activity because each of fusion proteins that disrupted centrosomes also localized to the nucleus.

## Conclusion

Based on our data, we conclude that the cellular roles of endogenous Nek1 likely include regulation of ciliogenesis, possibly via effects on centrosome remodelling. We interpret the results of this study with caution because they are based exclusively on over-expression phenotypes of tissue culture cells. Nevertheless, the pattern of phenotypes caused by the array of mutant forms of Nek1 used in this work is informative. Our data strongly indicate that the phenotypes are related to Nek1 kinase activity. Furthermore, the phenotypes are specific to over-expression of mNek1. For example, over-expression of mNek8 does not affect ciliogenesis, centrosomes or cell cycle progression [43,44]. Therefore, from the current work we conclude that an excess of Nek1 kinase activity inhibits assembly of cilia. Considering our data in the context of other studies (see Discussion) we further speculate that Nek1 participates in signalling between the primary cilium and the nucleus and effects the coordination of ciliogenesis with cell cycle progression.

## Methods

### Cell lines, cell culture and microscopy

The murine renal epithelial cell line derived from inner medullary collecting duct (IMCD3) was maintained in a 1:1 mixture of DMEM and Ham's F12 medium supplemented with 10% fetal bovine serum (DMEM-F12 (+), Invitrogen, Carlsbad, CA). Experiments were performed using cells below passage ten. Transient transfections were carried out with 4 μg of plasmid using Lipofectamine 2000 (Invitrogen) in OPTI-MEM reduced-serum media, according to the manufacturer's protocol. After 6 hours, cells were washed and transferred to pre-warmed DMEM-F12 (+). We set this as time zero post-transfection. For immunofluorescence, cells grown on coverslips were fixed with -20°C methanol for 10 minutes and rehydrated in PBS. The coverslips were incubated in primary antibody diluted in PBS for 1 hour, followed by two 15-minute PBS washes. The coverslips were then incubated in secondary antibody for 1 hour, followed by two 15-minute PBS washes. Nuclei were stained by a 10 minute, 1 μg/mL 4', 6-diamidino-2-phenylindole (DAPI) incubation at room-temperature. Stained coverslips were mounted in mowiol (Calbiochem, Dan Diego, CA). Immunofluorescence microscopy was performed using the Delta Vision system (Applied Precision, Isaaquah, WA) as described previously [8].

### Antibodies

The primary antibodies used include mouse monoclonal anti-γ tubulin (clone GTU-88; 1:1000; Sigma-Aldrich, St. Louis, MO), rabbit polyclonal anti-γ tubulin (1:1000; Sigma-Aldrich), mouse monoclonal anti-acetylated tubulin (clone 6-11B-1; 1:2000; Sigma-Aldrich), mouse monoclonal anti-myc (Clone 9E10; 1:2000; Clontech, Palo Alto, CA), mouse monoclonal anti-α-tubulin-FITC conjugate (Clone DM1A; 1:1000; Sigma-Aldrich), mouse monoclonal anti-centrin (1:1000; from J. Salisbury, Mayo Clinic, Rochester, MN; [45]), mouse monoclonal anti-PCNA (1:200; Calbiochem), rabbit polyclonal anti-mouse cleaved PARP (1:100; Cell Signaling Technologies, Danvers, MA), rabbit polyclonal anti-mNek1 (1:100; from Dr. Y. Chen, The University of Texas Health Science Center, San Antonio), human autoimmune serum M4491 (which is reactive to multiple components of the proximal pericentrosomal tube, [46]; 1:3000; from J. Rattner, University of Calgary, Alberta), rabbit polyclonal anti-pericentrin (1:1000; Covance Research Products, Denver, PA) and rabbit polyclonal anti-BBS6 (1:200; from M. Leroux, Simon Fraser University, Burnaby, BC; [47]). The secondary antibodies used include Alexa Fluor 488-conjugated goat anti-rabbit IgG (1:500; Molecular Probes, Eugene, OR), Alexa Fluor 594-conjugated goat anti-human IgG (1:1000; Molecular Probes), Alexa Fluor 594-conjugated goat anti-mouse IgG (1:2000; Molecular Probes) and Cy5-conjugated goat anti-rabbit IgG (1:500; Southern Biotech, Birmingham, AL).

### Molecular Constructs

mNek1 (accession number AY850065) was cloned into the SacI-SalI sites of pEGFP-C2 (Clontech), and truncations were made using standard molecular biology techniques. To generate myc-mNek1, the full-length mNek1 sequence was cloned into the Sal1 and Not1 sites of the pCMV-myc plasmid.

## Authors' contributions

MCW performed the experiments, helped to draft the manuscript and participated in the design of the study. LMQ conceived the study, participated in its design and drafted the manuscript. Both authors have read and approved the final manuscript.

## References

[B1] Lewtin K, Mizzen L, Motro B, Ben-David Y, Bernstein A, Pawson T (1992). A mammalian dual specificity protein kinase, Nek1, is related to the NIMA cell cycle regulator and highly expressed in meiotic germ cells. EMBO J.

[B2] Janaswami PM, Birkenmeier EH, Cook SA, Rowe LB, Bronson RT, Davisson MT (1997). Identification and genetic mapping of a new polycystic kidney disease on mouse chromosome 8. Genomics.

[B3] Vogler C, Homan S, Pung A, Thorpe C, Barker J, Birkenmeier EH, Upadhya P (1999). Clinical and pathological findings in two new allelic murine models of polycystic kidney disease. J Am Soc Nephrol.

[B4] Upadhya P, Birkenmeier EH, Birkenmeier CS, Barker JE (2000). Mutations in a NIMA-related kinase gene, Nek1, cause pleiotropic effects including a progressive polycystic kidney disease in mice. PNAS.

[B5] Arama E, Yanai A, Kilfin G, Bernstein A, Motro B (1998). Murine NIMA-related kinases are expressed in patterns suggesting distinct functions in gametogenesis and a role in the nervous system. Oncogene.

[B6] O'Connell MJ, Krien MJE, Hunter T (2003). Never say never. The NIMA-related protein kinases in mitotic control. Trends Cell Biol.

[B7] Mahjoub MR, Montpetit B, Zhao L, Finst RJ, Goh B, Kim AC, Quarmby LM (2002). The FA2 gene of Chlamydomonas encodes a NIMA family kinase with roles in cell cycle progression and microtubule severing during deflagellation. J Cell Sci.

[B8] Mahjoub MR, Rasi MQ, Quarmby LM (2004). A NIMA-related kinase, Fa2p, localizes to a novel site in the proximal cilia of Chlamydomonas and mouse kidney cells. Mol Biol Cell.

[B9] Bradley BA, Quarmby LM (2005). A NIMA-related kinase, Cnk2p, regulates both flagellar length and cell size in Chlamydomonas. J Cell Sci.

[B10] Quarmby LM, Mahjoub MR (2005). Caught Nek-ing: cilia and centrioles. J Cell Sci.

[B11] Parker JD, Bradley BA, Mooers AO, Quarmby LM (2007). Phylogenetic analysis of the neks reveals early diversification of ciliary-cell cycle kinases. PLoS ONE.

[B12] Simons M, Walz G (2006). Polycystic kidney disease: cell division without a c(l)ue?. Kidney Int.

[B13] Pazour GJ (2004). Intraflagellar transport and cilia-dependent renal disease: the ciliary hypothesis of polycystic kidney disease. J Am Soc Nephrol.

[B14] Mahjoub MR, Trapp ML, Quarmby LM (2005). NIMA-related kinases defective in murine models of polycystic kidney diseases localize to primary cilia and centrosomes. J Am Soc Nephrol.

[B15] Lupas A, Van Dyke M, Stock J (1991). Predicting coiled coils from protein sequences. Science.

[B16] Cokol M, Nair R, Rost B (2000). Finding nuclear localization signals. EMBO Rep.

[B17] La Cour T, Kiemer L, Molgaard A, Gupta R, Skriver K, Brunak S (2004). Analysis and prediction of leucine-rich nuclear export signals. Protein Eng Des Sel.

[B18] Zabner J, Fasbender AJ, Moninger T, Poellinger KA, Welsh MJ (1995). Cellular and molecular barriers to gene transfer by a cationic lipid. J Biol Chem.

[B19] Wilke M, Fortunati E, Broek M van den, Hoogeveen AT, Scholte BJ (1996). Efficacy of a peptide-based gene delivery system depends on mitotic activity. Gene Therapy.

[B20] Tseng W, Haselton FR, Giorgio TD (1999). Mitosis enhances transgene expression of plasmid delivered by cationic liposomes. Biochim Biophys Acta.

[B21] Mortimer I, Tam P, MacLachlan I, Graham RW, Saravolac EG, Joshi PB (1999). Cationic lipid-mediated transfection of cells in culture requires mitotic activity. Gene Ther.

[B22] Quarmby LM, Parker JD (2005). Cilia and the cell cycle?. J Cell Biol.

[B23] Hanks SK, Hunter T (1995). The eukaryotic protein kinase superfamily: kinase (catalytic) domain structure and classification. FASEB J.

[B24] Noguchi K, Fukazawa H, Murakami Y, Uehara Y (2002). Nek11, a new member of the NIMA family of kinases, involved in DNA replication and genotoxic stress responses. J Biol Chem.

[B25] Twomey C, Wattem SL, Pillai MR, Rapley J, Baxter JE, Fry AM (2004). Nek2B stimulates zygotic centrosome assembly in Xenopus laevis in a kinase-independent manner. Dev Biol.

[B26] Richardson A, Malik RK, Hildebrand JD, Parsons JT (1997). Inhibition of cell spreading by expression of the C-terminal domain of focal adhesion kinase (FAK) is rescued by coexpression of Src or catalytically inactive FAK: a role for paxillin tyrosine phosphorylation. Mol Cell Biol.

[B27] Jang YJ, Lin CY, Ma S, Erikson RL (2002). Functional studies on the role of the C-terminal domain of mammalian polo-like kinase. Proc Natl Acad Sci.

[B28] Surpili MJ, Delben TM, Kobarg J (2003). Identification of proteins that interact with the central coiled-coil region of the human protein kinase NEK1. Biochemistry.

[B29] Feige E, Shalom O, Tsuriel S, Tissachar N, Motro B (2006). Nek1 shares structural and functional similarities with NIMA kinase. Biochim Biophys Acta.

[B30] Paoletti A, Moudjou M, Paintrand M, Salisbury JL, Bornens M (1996). Most of centrin in animal cells is not centrosome-associated and centrosomal centrin is confined to the distal lumen of centrioles. J Cell Sci.

[B31] Wassem NH, Lane DP (1990). Monoclonal antibody analysis of the proliferating cell nuclear antigen (PCNA) Structural conservation and the detection of a nucleolar form. J Cell Sci.

[B32] Mikule K, Delaval B, Kaldis P, Jurcyzk A, Hergert P, Doxsey S (2007). Loss of centrosome integrity induces p38-p53-p21-dependent G1-S arrest. Nat Cell Biol.

[B33] Kudo N, Wolff B, Sekimoto T, Schreiner EP, Yoneda Y, Yanaqida M, Horinouchi S, Yoshida M (1998). Leptomycin B inhibition of signal-mediated nuclear export by direct binding to CRM1. Exp Cell Res.

[B34] Badano JL, Mitsuma N, Beales PL, Katsanis N (2006). The ciliopathies: and emerging class of human genetic disorders. Annu Rev Genomics Hum Genet.

[B35] Avner ED (1993). Epithelial polarity and differentiation in polycystic kidney disease. J Cell Sci Suppl.

[B36] Woo D (1995). Apoptotsis and loss of renal tissue in polycystic kidney diseases. N Engl J Med.

[B37] Fischer E, Leque E, Doyen A, Nato F, Nicolas JF, Torres V, Yaniv M, Pontoglio M (2006). Defective planar cell polarity in polycystic kidney disease. Nat Genet.

[B38] Caspary T, Larkins CE, Anderson KV (2007). The graded response to Sonic Hedgehog depends on cilia architecture. Dev Cell.

[B39] Christensen ST, Pedersen LB, Schneider L, Satir P (2007). Sensory cilia and integration of signal transduction in human health and disease. Traffic.

[B40] Wemmer KA, Marshall WF (2007). Flagellar length control in *Chlamydomonas *– paradigm for organelle size regulation. Int Rev Cytol.

[B41] Wloga D, Camba A, Rogowski K, Manning G, Dziadosz M, Gaertig J (2006). Members of NIMA-related kinase family promote disassembly of cilia by multiple mechanisms. Mol Biol Cell.

[B42] Pugacheva EN, Jablonski SA, Hartman TR, Henske EP, Golemis EA (2007). HEF1-dependent Aurora A activation induces disassembly of the primary cilium. Cell.

[B43] Trapp ML, Galtseva A, Manning DK, Beier DR, Rosenblum ND, Quarmby LM Defects in ciliary localization of Nek8 in a subset of kidney tubules is associated with cystogenesis. Paediatric Nephrol.

[B44] Otto EA, Trapp ML, Schultheiss UT, Quarmby LM, Hildebrandt F (2007). Mutations in NIMA-related kinase NEK8 causes nephronophthisis in humans and affects ciliary and centrosomal localization. J Am Soc Nephrol.

[B45] Baron AT, Geenwood TM, Bazinet CW, Salisbury JL (1992). Centrin is a component of the pericentriolar lattice. Biology of the Cell.

[B46] Ou Y, Ratner JB (2000). A subset of centrosomal proteins are arranged in a tubular conformation that is reproduced during centrosome duplication.. Cell Motil Cytoskeleton.

[B47] Kim JC, Ou Y, Badano JL, Esmail MA, Leitch CC, Fiedrich E, Beales PL, Archibald JM, Katsanis N, Rattner JB, Leroux MR (2005). MKKS/BBS6, a divergent chaperonin-like protein linked to the obesity disorder Bardet-Biedl syndrome, is a novel centrosomal component required for cytokinesis. J Cell Sci.

